# Risk factors for anastomotic complications after one-stage anastomosis for oesophageal atresia

**DOI:** 10.1186/s13019-021-01557-0

**Published:** 2021-06-19

**Authors:** Jin-Xi Huang, Song-Ming Hong, Qiang Chen, Zeng-Chun Wang, Dian-Ming Wu, Jun-Jie Hong, Chaoming Zhou

**Affiliations:** 1 Department of Cardiothoracic Surgery, Fujian Maternity and Child Health Hospital, Affiliated Hospital of Fujian Medical University, Fuzhou, China; 2 Fujian Key Laboratory of Women and Children’s Critical Diseases Research, Fujian Maternity and Child Health Hospital, Fuzhou, China; 3 Fujian Branch of Shanghai Children’s Medical Center, Fuzhou, China; 4 Fujian Children’s Hospital, Fuzhou, China

**Keywords:** Oesophageal atresia, One-stage anastomosis, Anastomotic fistula, Anastomotic stricture, Risk factor analysis

## Abstract

**Background:**

Oesophageal atresia is a congenital malformation of the oesophagus and a serious malformation of the digestive system, postoperative complications include acute respiratory failure, pneumonia, anastomotic fistula, anastomotic stenosis, tracheal stenosis, gastroesophageal reflux and eosinophilic oesophagitis, anastomotic fistula is one of the important causes of postoperative death. The objective of this study is to identify the risk factors for anastomotic complications after one-stage anastomosis for oesophageal atresia.

**Methods:**

A retrospective analysis was performed on the clinical data of 107 children with congenital oesophageal atresia who underwent one-stage anastomosis in our hospital from January 2013 to December 2018. Single-factor and multivariate logistic regression analyses were performed to determine the risk factors for anastomotic fistula and anastomotic stenosis.

**Results:**

A total of 107 children with oesophageal atresia underwent one-stage anastomosis, and the incidence of anastomotic fistula was 26.2%. The probability of anastomotic stenosis in the long term was 52.3%, and the incidence of refractory stenosis (dilation ≥5 times) was 13.1%. Analysis of the clinical count data in the anastomotic fistula group and non-anastomotic fistula group showed that preoperative albumin (F = 4.199, *P* = 0.043), low birth weight (F = 7.668, *P* = 0.007) and long gap defects (F = 6.107, *P* = 0.015) were risk factors for postoperative anastomotic fistula. Further multivariate logistic regression analysis showed that low birth weight (Wald2 = 4.499, *P* = 0.034, OR = 2.775) and long gap defects (Wald2 = 6.769, *P* = 0.009, OR = 4.939) were independent risk factors for postoperative anastomotic fistula. Premature delivery (F = 5.338, *P* = 0.023), anastomotic fistula (F = 11.381, *P* = 0.001), endoscopic surgery (F = 6.343, *P* = 0.013), preoperative neutrophil count (F = 8.602, *P* = 0.004), preoperative low albumin (F = 8.410, *P* = 0.005), and a preoperative prognostic nutritional index < 54 (F = 5.54, *P* = 0.02) were risk factors for refractory anastomotic stenosis in children. Further multivariate logistic regression analysis showed that postoperative anastomotic fistula (Wald2 = 11.417, *P* = 0.001, OR = 8.798), endoscopic surgery (Wald2 = 9.633, *P* = 0.002, OR = 4.808), and a prognostic nutritional index < 54 (Wald2 = 4.540, P = 0.002, OR = 2.3798) were independent risk factors for refractory anastomotic stenosis.

**Conclusion:**

Low birth weight and long gap defects are important predictors of postoperative anastomotic fistula, and the possibility of refractory anastomotic stenosis should be considered. The long-term risk of anastomotic stenosis was increased in children undergoing endoscopic surgery and in those with a preoperative prognostic nutritional index < 54.

## Background

Oesophageal atresia (EA) is a congenital malformation of the oesophagus and a serious malformation of the digestive system. The global overall incidence is 2.4 cases per 100,000, and the incidence of congenital EA in China is 1/1250–1/4000 [1]. At present, this disease is believed to be due to the interruption of oesophageal continuity caused by abnormal development of the preembryonic intestinal area. Approximately 70–90% of children often have tracheal oesophageal fistula (TEF) [2], and 55% of children may have other systemic congenital malformations, especially severe heart malformations, which is an important factor for poor prognosis in children [3]. The treatment of EA has made great progress in the past 20 years, and the survival rate can reach more than 90%. With the development of endoscopic technology in recent years, video-assisted one-stage anastomosis has gradually become the main surgical method instead of open-chest operations.

Common EA postoperative complications include acute respiratory failure, pneumonia, anastomotic fistula, anastomotic stenosis, tracheal stenosis, gastroesophageal reflux and eosinophilic oesophagitis [4]. Anastomotic complications mainly include recent postoperative anastomotic fistula and refractory anastomotic stenosis, and anastomotic fistula is one of the important causes of postoperative death. Severe anastomotic fistula may increase the risk of refractory anastomotic stenosis [5]. This study attempts to explore the related risk factors for postoperative anastomotic complications in children with congenital EA through a retrospective study.

## Methods

Excluded two infants with EA type Vogt II and another with IV, data from the 127 children born in or admitted to our hospital with EA type Vogt IIIb were collected and followed, with 14 of the parents of sick children refusing surgery. A total of 113 children received surgical treatment, and 6 died perioperatively; thus, 107 children were enrolled. The patients were divided into two groups according to the occurrence of anastomotic fistula.

### General conditions

From January 2013 to December 2018, 107 children with EA underwent surgery in our hospital with complete clinical and pathological data; there were 74 male patients and 33 female patients. The data of sex, birth weight, gestational age, surgical method, defect length (intraoperative measurement), preoperative mechanical ventilation, and combined deformity were collected (Table 1).

**Table 1:** General Information

Information types		Value
Gender (male/female)		74/33
Gestational weeks		38.51 ± 1.61
Birth weight (kg)		2.83 ± 0.48
Operation method (endoscopic/open)		49/58
Preoperative mechanical ventilation (yes/no)		7/100
Accompanying anomalies		33/74
Cardiovascular anomalies		14
	Tetralogy of fallot	1
	Ventricular septal defect	7
	Atrial septal defect	6
Gastrointestinal anomalies		8
	Malrotationof intestine	1
	Intestinal atresia	1
	Hypertrophic pyloric stenosis	1
	Anal atresia	3
	Hirschsprung’s disease	2
Genitourinary anomalies anomalies		4
	Polycystic kidney	1
	Vesico-ureteral reflux	1
	UPJO	2
Musculoskeletal anomalies		11
	Hemivertebra deformity	3
	Congenital absence of rib	3
	Syndactylia	5
Respiratory anomalies		11
	Tracheostenosis	1
	Subglottic stenosis	3
	Tracheomalacia	5
	Congenital cystic lung disease	1
	Bronchopulmonary dysplasia	1
Other anomalies		3
	Connatal cyst	1
	Congenital microtia	1
	Dandy-Walker syndrome	1
VACTERL association		23
Albumin (g/L)		34.16 ± 3.2
Neutrophil (10*9/ L)		9.97 ± 4.89
lymphocyte (10*9/ L)		3.5 ± 1.52
NLR		3.28 ± 1.88
Albumin/Globulin		1.87 ± 0.71
PNI		51.69 ± 8.46
Gap (cm)		1.53 ± 0.96
Anastomotic fistula		28
Anastomotic stenosis		56

### Preoperative data

1. Serological indicators: the preoperative neutrophil count, preoperative lymphocyte count, and neutrophil to lymphocyte ratio (NLR).
2. Nutritional indicators: the preoperative albumin, prognostic nutritional index (PNI) and albumin to globulin ratio. PNI = 10ALB(dg/L) + 5*LYMPH#.

### Key operative steps

#### Open surgery

Right posterolateral thoracotomy, extrapleural approach to the posteromedial mediastinum, to expose the TEF, and futher ligation is undertaken using Hem-o-Lok clips (Sinolinks clips; Sinolinks Medical Innovation,inc. Jiangsu, China). Following TEF ligation, upper pouch is dissected and incised in readiness for anastomosis. Oesophageal anastomosis with fine interrupted sutures begins with the back wall, following which a trans-anastomotic nasogastric tube is passed prior to completion of the anastomosis of the front wall.

#### Thoracoscopic surgery

The first step is to identify the TEF. The TEF is dissected and ligated using Hem-o-Lok clips. The upper pouch is identified and dissected with the aid of gentle pressure on the 10 Fr tube. The anastomosis is performed using interrupted sutures secured by intracorporeal ties. Once complete, a chest tube may be left through one of the port site incisions.

### Postoperative data

Anastomotic fistula, anastomotic stenosis, pulmonary infection, oesophageal dilatation times and first dilatation time.

### Inclusion criteria

Children born or admitted to our hospital with congenital EA and receiving stage I anastomosis with complete clinical data. The exclusion criteria were as follows: 1. parents refused the operation; 2. patients who could not undergo delayed anastomosis in the first stage of the operation; 3. patients who died in the perioperative period.

1.6 SPSS 22.0 software was used to analyse the data, and univariate and multivariate logistic regression analyses were performed for EA anastomotic fistula and anastomotic stenosis. The odds ratio (OR) and 95% confidence interval (95% CI) were used to indicate the correlation. T tests were used for the comparison of oesophageal dilatation analysis results, and *P* < 0.05 was considered statistically significant.

## Results

### Recent anastomotic complications: anastomotic fistula

Anastomotic fistula is a common and dangerous recent anastomotic complication of oesophageal reconstruction. In this study, the incidence of anastomotic fistula was 26.3%, which is slightly higher than the 20% reported in other studies [6]. The occurrence of anastomotic fistula after anastomosis in adults is mainly related to anastomotic tension and anastomotic blood supply [7]. It is difficult to quantitatively evaluate anastomotic tension and anastomotic blood supply during neonatal EA stage I anastomosis. In this study, clinical enumeration data were collected to seek objective clinical indicators to assess the risk of anastomotic complications. With the analysis of two groups of data, continuous variables using single-factor analysis of variance and classification variables using the chi-square test, the study found that preoperative albumin, birth weight and defect length were postoperative anastomotic fistula-related factors. The differences between groups were statistically significant, and the time, gestational age, sex, preoperative operation method of mechanical ventilation, neutrophil count, lymphocyte count, NLR, PNI, albumin, globulin, and merged heart deformities were not significantly different between these factors in the two groups. Further multivariate logistic regression analysis showed that a birth weight less than 2500 g and long gap defects larger than 3 cm were independent risk factors for postoperative anastomotic fistula in children. (Table 2 & Table 3).

**Table 2:** Comparison of complications between anastomotic fistula group and non-anastomotic fistula group

Factors		Anastomotic fistula	Non-anastomotic fistula	F/χ2	P
Gestational weeks		38.61 ± 1.61	38.32 ± 1.63	0.651	0.421
Birth weight (kg)		2.62 ± 0.51	2.90 ± 0.47	7.668	0.007
Gap (cm)		1.91 ± 1.01	1.39 ± 0.91	6.107	0.015
Albumin (g/L)		33.11 ± 3.04	34.54 ± 3.19	4.199	0.043
Neutrophil (10*9/ L)		10.29 ± 5.34	9.85 ± 4.75	0.174	0.678
Lymphocyte(10*9/L)		3.69 ± 1.57	3.44 ± 1.50	0.583	0.447
NLR		3.13 ± 1.89	3.30 ± 1.88	0.15	0.7
PNI		51.59 ± 7.67	51.73 ± 8.78	0.006	0.938
Albumin/Globulin		2.03 ± 1.23	1.81 ± 0.38	2.026	0.158
Accompanying anomalies	Yes	12	21	2.567	0.088
	No	16	58		
Cardiovascular anomalies	Yes	4	10	0.635	0.316
	No	24	69		
Preoperative mechanical ventilation	Yes	1	6	0.547	0.41
	No	27	73		
Gender	Male	18	56	0.422	0.336
	Female	10	23		
Operation method	Open	15	43	0.006	0.055
	VATS	13	36		
VACTERL association	Yes	6	17	0.992	0.610
	No	22	62

**Table 3:** Multiple factor logistic regression

Factors	Waldχ2	*P* values	OR
Low birth weight (less than 2500 g)	4.499	0.004	2.775
long gap atresia (larger than 3 cm)	6.769	0.034	4.939

### Refractory anastomotic complications: anastomotic stenosis

Anastomotic stricture: symptoms of dysphagia and anastomotic stricture were confirmed by gastrointestinal radiography or endoscopy. This study found that the surgical method, gestational age, preoperative neutrophil count, PNI and anastomotic fistula may be related factors for the occurrence of anastomotic stenosis. Further multivariate logistic regression analysis found that open surgery, anastomotic fistula and low nutritional status (PNI ≤54) were high-risk factors for refractory anastomotic stenosis (Table 4 & Table 5).

**Table 4:** Univariate analysis of anastomotic stenosis

Factors		Anastomotic stenosis	Non-anastomotic stenosis	F/χ2	P
Gestational weeks		38.20 ± 1.73	38.90 ± 1.39	5.338	0.023
Birth weight (kg)		2.77 ± 0.52	2.90 ± 0.44	1.882	0.173
Gap (cm)		1.60 ± 0.93	1.46 ± 0.99	0.546	0.462
Neutrophil (10*9/ L)		8.69 ± 4.69	11.37 ± 4.77	8.602	0.004
Lymphocyte (10*9/L)		3.31 ± 1.28	3.72 ± 1.73	1.947	0.166
NLR		3.00 ± 2.05	3.53 ± 1.64	2.097	0.151
PNI		49.9 ± 6.97	53.67 ± 9.53	5.54	0.02
Albumin/Globulin		1.77 ± 0.35	1.98 ± 0.95	2.584	0.112
Accompanying anomalies	Yes	17	16	0.013	0.538
	No	39	35		
Cardiovascular anomalies	Yes	8	6	0.149	0.462
	No	48	45		
Preoperative mechanical ventilation	Yes	4	3	0.69	0.552
	No	52	48		
Gender	Male	39	35	0.013	0.538
	Female	17	16		
Anastomotic fistula	Yes	22	6	10.464	0.001
	No	34	45		
Operation method	Open	24	34	6.096	0.013
	VATS	32	17		
VACTERL association	Yes	11	12	0.986	0.585
	No	40	44

**Table 5:** Logistic regression analysis results

Factors	Waldχ2	P values	OR(95%CI)
Open operation	5.106	0.025	2.993 (1.147–7.815)
Anastomotic fistula	10.204	0.01	7.506 (2.179–25.856)
PNI ≤ 54	7.829	0.005	5.725 (1.686–19.434)

## Discussion

### Risk factor analysis of recent anastomotic fistula

The occurrence of anastomotic fistula after first-stage oesophagostomy in children with congenital EA is still a serious complication that troubles surgeons and has an important influence on the prognosis and quality of life of children [8]. The occurrence of anastomotic fistula is mainly related to anastomotic blood supply, anastomotic tension and other factors [9, 10]. The incidence of anastomotic fistula after end-to-end oesophagostomy is reported to be approximately 8–12% in adults [11], and the incidence of anastomotic fistula after EA is approximately 20% [3]. This may be related to the large anastomotic tension and poor anastomotic blood supply caused by oesophageal defects. In this study, univariate analysis found that albumin, defect length and birth weight may be related to the occurrence of anastomotic fistula. Multivariate logistic regression analysis found that long gap defects and low birth weight were high risk factors for anastomotic fistula.

Preoperative low protein may lead to postoperative anastomotic oedema patients, which increases anastomotic tension and leads to poor local healing [12, 13]. However, in multiple factor analysis, albumin levels were not included in the regression equation. This may be because, in this study, the number of patients with low albumin (albumin < 28 g/L) was less relevant, further expanding the sample size to find the best values for a more suitable cut-off value to evaluate the relationship between albumin levels and anastomotic fistula.

The defect length is one of the important indexes for the preoperative evaluation of operation difficulty in children with EA [14]. In this study, the mean defect length was 1.53 ± 0.96 cm, 1.91 ± 1.01 cm in children with anastomotic fistula, and 1.39 ± 0.91 cm in children without anastomotic fistula, showing statistically significant differences. There were 14 patients with long gap EA, and the possibility of anastomotic fistula was 4.9 times that of non-long-gap EA. Long defects are an independent risk factor for anastomotic fistula. Children with an intraoperative defect length > 3 cm were more prone to anastomotic fistula 7 to 10 days after surgery. Four cases were excluded from this study due to intrathoracic extension and delayed anastomosis of long gap EAs. Fourteen cases of long gap EAs appeared among 8 cases of severe anastomotic fistula. We believe that the use of long gap defects to extend the anastomosis may be a better choice.

Birth weight reflects the developmental level of children in utero, and a low birth weight is often associated with a history of premature birth and foetal distress [15]. Children with a low birth weight may have more oesophageal defects due to the short duration of natural extension of the oesophagus in utero, and these defects may be accompanied by poorer peripheral circulation and lower cardiac output [16], which may lead to poor local blood supply at the anastomosis. The counting data showed that 8 of 32 children with a low birth weight had heart malformations, while only 6 of 75 children with a normal weight had heart malformations. This study found that the risk of anastomotic fistula was 2.7 times higher in children with a low birth weight than in children with a normal weight.

### Risk factor analysis of refractory anastomotic stenosis

European Society of Gastrointestinal Endoscopy (ESGE) and European Society for Paediatric Gastroenterology Hepatology and Nutrition (ESPGHAN) suggest the following definition of a benign refractory stricture in children: After 5 dilation sessions with maximal 4-week intervals, there is an inability to successfully remediate the anatomic problem to obtain age-appropriate feeding possibilities [17]. In this study, the probability of anastomotic stenosis was 52.3%, and the risk of anastomotic stenosis factors, including anastomotic fistula, long defects, severe gastroesophageal reflux and eosinophilic oesophagitis [18, 19], may cause inflammation of the anastomotic fistula after conservative treatment as well as related scar factors. Anastomotic fistula was one of the most relevant factors, OR = 7.506, but the length of the defect was not included in the regression equation. Univariate analysis revealed that the surgical method, gestational age, preoperative neutrophil count, and PNI may also be related factors. In multivariate analysis, thoracoscopic surgery, anastomotic fistula, and low nutritional status (PNI ≤54) were high risk factors for anastomotic stenosis. The incidence of anastomotic stenosis after thoracoscopic surgery was 32/49, which was significantly higher than that after open surgery (24/58). Open surgery may provide better oesophageal mucosal alignment and firmer end-to-end anastomosis. Thoracoscopic oesophageal anastomosis is still under development, and it is believed that the incidence of anastomotic stenosis in endoscopic surgery will decrease with the maturity of endoscopic technology in the future. According to the results of this study, thoracoscopic surgery is still considered to increase the risk of anastomotic stenosis.

Most anastomotic fistulas can be cured by conservative treatment, including total parenteral nutrition support or post-pyloric enteral nutrition support, pleural drainage and adequate antibiotic use [20, 21]. Some scholars advocate early operations to close the fistula in order to avoid severe pulmonary infection and empyema [22, 23]. Anastomotic fistula leads to severe anastomotic inflammation. Although conservative treatment is successful, it is still inevitable that the incidence of anastomotic stenosis will increase or even develop into refractory anastomotic stenosis due to scarring [24].

At present, there are few studies on nutritional assessment indicators for neonates, and the PNI is a commonly used nutritional assessment indicator for gastrointestinal tumours in adults [25]. Univariate analysis found that the preoperative difference in PNI values between the two groups was statistically significant. At present, there is no clear numerical classification of neonatal nutritional risk with the PNI. By calculating the Youden index, we obtained the optimal truncation value of the PNI value in this sample to be 54. Based on this, we conducted a multifactor analysis and found that the risk of anastomotic stenosis in children in the low PNI group (PNI ≤54) was 5.7 times higher than that in the high PNI group. For children with preoperative nutritional risk, perioperative nutritional support may help reduce the risk of refractory anastomotic stenosis, which needs to be further confirmed by future prospective studies.

## Conclusion

Low birth weight and long gap defects are important predictors of postoperative anastomotic fistula, and the possibility of refractory anastomotic stenosis should be considered. The long-term risk of anastomotic stenosis was increased in children undergoing endoscopic surgery and in those with a preoperative prognostic nutritional index < 54.

## Data Availability

Written informed consent was obtained from the patients for publication of this article.

## Abbreviations

EA: Oesophageal atresia
TEF: Tracheal oesophageal fistula
NLR: Neutrophil to lymphocyte ratio
PNI: Prognostic nutritional index
OR: Odds ratio
CI: Confidence interval
VATS: Video-assisted thoracic surgery
UPJO: Uretero pelvic juriction obstruction
